# Novel Phage Lysin Abp013 against *Acinetobacter baumannii*

**DOI:** 10.3390/antibiotics11020169

**Published:** 2022-01-28

**Authors:** Joash Jun Keat Chu, Wee Han Poh, Nabilah Taqiah Binte Hasnuddin, En Yi Hew, Linh Chi Dam, Abbas El Sahili, Scott A. Rice, Boon Chong Goh

**Affiliations:** 1 Antimicrobial Resistance Interdisciplinary Research Group, Singapore-MIT Alliance for Research and Technology Centre, Singapore 138602, Singapore; jchu011@e.ntu.edu.sg (J.J.K.C.); s10177065@connect.np.edu.sg (E.Y.H.); LINHCHI001@e.ntu.edu.sg (L.C.D.); 2 School of Biological Sciences, Nanyang Technological University, Singapore 637551, Singapore; aelsahili@ntu.edu.sg; 3 Singapore Centre for Environmental Life Sciences Engineering, School of Biological Sciences, Nanyang Technological University, Singapore 637551, Singapore; whpoh@ntu.edu.sg (W.H.P.); nabilah.taqiah@ntu.edu.sg (N.T.B.H.); 4 The Ithree Institute, University of Technology Sydney, Sydney, NSW 2007, Australia

**Keywords:** phage lysin, endolysin, multidrug resistance, *Acinetobacter baumannii*, novel antibacterial agent

## Abstract

As antimicrobial resistance (AMR) continues to pose an ever-growing global health threat, propelling us into a post-antibiotic era, novel alternative therapeutic agents are urgently required. Lysins are bacteriophage-encoded peptidoglycan hydrolases that display great potential as a novel class of antimicrobials for therapeutics. While lysins against Gram-positive bacteria are highly effective when applied exogenously, it is challenging for lysins to access and cleave the peptidoglycan of Gram-negative bacteria due to their outer membrane. In this study, we identify a novel phage lysin Abp013 against *Acinetobacter baumannii*. Abp013 exhibited significant lytic activity against multidrug-resistant strains of *A. baumannii*. Notably, we found that Abp013 was able to tolerate the presence of human serum by up to 10%. Using confocal microscopy and LIVE/DEAD staining, we show that Abp013 can access and kill the bacterial cells residing in the biofilm. These results highlight the intrinsic bacteriolytic property of Abp013, suggesting the promising use of Abp013 as a novel therapeutic agent.

## 1. Introduction

The inappropriate and extensive use of antibiotics over the past few decades have led to the emergence of antimicrobial resistance (AMR) [1,2]. This phenomenon is particularly startling in Gram-negative bacteria, including multidrug-resistant (MDR) *Acinetobacter baumannii*, *Klebsiella pneumoniae*, and *Pseudomonas aeruginosa*, which are responsible for serious nosocomial infections that often lead to high rates of morbidity and mortality [3]. Antibiotics, which are indispensable for bacterial infection treatments, have become less effective against these MDR bacteria [4,5]. Thus, the decline in antibiotic efficacy and the lack of novel antibiotics have placed us closer to a post-antibiotic era, necessitating the prompt and immediate search as well as the development of novel alternative antimicrobial approaches to combat the serious threat posed by MDR bacterial infections [6].

Among the various alternative antibacterial agents, a promising approach involves the use of lysins. Lysins, also known as endolysins, are peptidoglycan hydrolases that hydrolyze crucial covalent bonds of the peptidoglycan layer required for maintaining the structural integrity of the cell wall [7]. Bacteriophages utilize lysins to penetrate the bacterial host during infection, and destroy the peptidoglycan to release the phage progeny during the phage lytic cycle [8]. Compared to conventional antibiotics, lysins offer numerous advantages, such as a lower probability of developing resistance [9], high specificity and selectivity for bacterial pathogens [10,11], and the ability to act on bacterial biofilms [12].

Previous studies have demonstrated the potential of utilizing recombinantly expressed lysins and applying them exogenously, a notion known as “lysis from without”. Generally, Gram-positive lysins are highly effective, resulting in the rapid lysis and death of the targeted bacteria due to a readily accessible peptidoglycan [13,14]. In contrast, the antimicrobial effects of exogenously added lysins are greatly diminished in Gram-negative bacteria, due to the presence of a protective outer membrane barrier that restricts the access and entry of lysins to the peptidoglycan through the bacterial lipopolysaccharide (LPS)-covered surfaces [15,16,17]. As such, few naturally occurring lysins have been reported to display antimicrobial activity against Gram-negative bacteria [15,18,19]. However, in recent years, studies reporting the simultaneous application of outer membrane permeabilizers (OMPs) and lysins, as well as the development of engineered lysins (Artilysins) through the modification of charged amino acid residues at their terminal region have shown improved antibacterial activity, overcoming the hindrance posed by the outer membrane [16,20].

While most lysins targeting Gram-positive bacteria are specific in targeting a single host species or genus, lysins against Gram-negative bacteria are typically found to be effective across multiple bacterial species [21,22]. The reason for their broad host-range can be ascribed to the cationic tail that facilities the penetration of lysins through the outer membrane of Gram-negative bacteria. Furthermore, some lysins targeting Gram-negative bacteria were found to have a non-enzymatic mechanism of action, such as antimicrobial peptide-like elements in the lysin sequences that aid in the destabilization of the outer membrane [23,24].

In this present study, we identify a novel phage lysin termed Abp013 against *A. baumannii*, and characterize it for its optimal dose concentration, pH, salt, and human serum tolerance. The lytic spectra of Abp013 are determined against a panel of MDR bacteria species, including *A. baumannii*, *K. pneumoniae*, and *P. aeruginosa*. Abp013 is also evaluated for its potential to kill bacteria residing inside a bacterial biofilm. The results obtained elucidate the intrinsic bacteriolytic property of Abp013, suggesting the promising use of Abp013 as a novel therapeutic agent. Lastly, our study discusses the potential in engineering Abp013 to create better lysins with higher specificity and selectivity against MDR Gram-negative bacteria.

## 2. Results

### 2.1. Identification and Bioinformatic Analysis of Abp013

**Figure 1:**
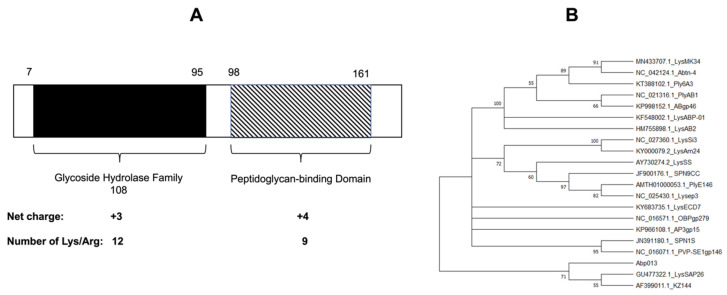
Bioinformatic analysis of Abp013. (**A**) Schematic diagram of the domain organization of Abp013. The glycoside hydrolase family 108 (net charge: +2) is located between amino acid residues 7 and 95, while the peptidoglycan-binding domain (net charge: +3) is located between residues 98 and 161. (**B**) Bootstrap consensus tree showing the phylogenetic relationship of Abp013 and previously reported Gram-negative lysins. The nucleotide sequences were aligned via ClustalW, and the evolutionary history was inferred via the Neighbor–Joining tree method using MEGA11 software.

The gene of lysin Abp013 was identified from the genome of an *A. baumannii* bacteriophage, φAbp2, and it was annotated as a secretion activator protein (ABP2_013) under the accession number MF346584.1 [25]. The analysis of the protein sequence using the NCBI Conserved Domain Database (CDD) and Simple Modular Architecture Research Tool (SMART) [26], revealed a glycoside hydrolase family 108 at the N-terminal, while a peptidoglycan-binding domain was located at the C-terminal (Figure 1A). The confidently predicted domains analyzed via SMART are listed in Supplementary Table S1.

A phylogenetic analysis utilizing the ClustalW alignment of Abp013 with other previously reported Gram-negative lysins showed a low similarity with other reported lysins (Figure 1B). As LysSAP26 and KZ144 share a common ancestor with Abp013, further analysis via the MUSCLE multiple alignment tool [27] revealed a low level of similarity of Abp013 with LysSAP26 and KZ144, suggesting the novelty of Abp013 (Supplementary Figure S1).

### 2.2. Expression, Purification, and Functional Characterization of Abp013

#### 2.2.1. Expression and Purification of Abp013

**Figure 2:**
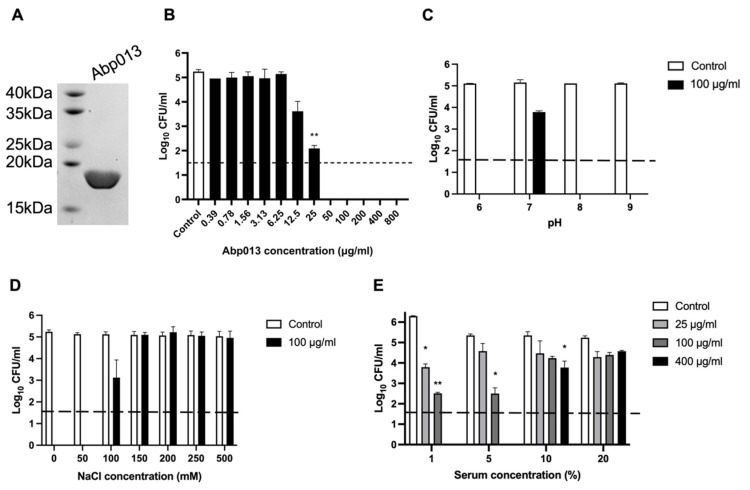
Purification of Abp013. (**A**) SDS-PAGE (12%) analysis of Abp013. Gel shows a highly purified 19.5 kDa Abp013 containing a C-terminal 6x HIS needed for affinity chromatography purification. Characterization of Abp013. The dose–response of Abp013 was determined (**B**) and the lytic activity of Abp013 was tested at various pH buffers (**C**) and under different salt concentrations (**D**). The tolerance of Abp013 towards human serum were tested as well (**E**). The number of log10 CFU/mL was determined through plating 10-fold serial dilution in a bactericidal assay and compared with buffer-treated negative controls. Statistical significance was determined by a two-tailed Student’s *t*-test with Welch’s correction. * *p* < 0.05; ** *p* < 0.01. The dashed line indicates the limit of detection. The experiments were carried out in biological duplicates; error bars represent the standard deviation.

The gene of Abp013 was cloned into a pNIC-CH vector, transformed in the BL21(DE3)-T1R *Escherichia coli* Rosetta strain. The Abp013 protein was purified via affinity chromatography using Ni-charged magnetic beads. The SDS-PAGE showed a single band slightly below the 20 kDa ladder, consistent with the estimated molecular mass of 19.5 kDa (Figure 2A). A high yield of 26 mg of purified Abp013 was obtained from a 1 L culture, which could be attributed to the optimization of the codon usage of the recombinant gene for *E. coli* expression.

#### 2.2.2. Dose Response of Abp013

To determine the optimal dose concentration of Abp013 for the subsequent experiments, the lytic activity of Abp013 (0.39 μg/mL to 800 μg/mL) was measured against *A. baumannii* ATCC 17961 by performing a bactericidal assay. At 25 μg/mL, Abp013 was able to achieve >90% reduction in log CFU, while concentrations beyond 25 μg/mL showed maximal lytic activity and achieved full log-CFU reduction in the number of viable bacterial cells (Figure 2B).

#### 2.2.3. Abp013 Characterization at Various pH and Salt Concentrations

The characterization of the bactericidal activity of Abp013 at different pH levels at 100 μg/mL, showed that Abp013 had high killing activity at pH 6.0, 8.0, and 9.0 (Figure 2C). While, at pH 7.0, there was a marked reduction in the killing activity, achieving only a ~1.3-log CFU reduction. This assay was repeated at a low lysin concentration of 10 µg/mL and it revealed both pH 6 and 8 are optimal (Supplementary Figure S2). pH 6.0 was chosen for the subsequent assays because it is a physiologically relevant pH for *A. baumannii* [28,29].

We then examined the halotolerance of Abp013 and the effects of varying NaCl concentrations on its killing efficacy. The log-phase *A. baumannii* cells were incubated with 100 μg/mL of Abp013 in buffers containing 0–500 mM of NaCl. The bacterial viability remained relatively constant throughout all the tested NaCl concentrations. The maximal killing activity of Abp013 was observed at 50 mM NaCl, and the lytic activity was significantly reduced when the concentration was raised to 100 mM NaCl (2-log CFU reduction) (Figure 2D). The subsequent increase in NaCl concentrations beyond 100 mM abolished the killing activity of Abp013.

#### 2.2.4. Efficacy of Abp013 in Serum

A challenge in the application of Gram-negative lysins is the abolished killing activity in the presence of human serum. The activity of Abp013 was tested in different concentrations (1%, 5%, 10%, and 20%) of human serum. Abp013 retained good bactericidal activity 1% serum, where Abp013 was able to achieve over 2-log CFU reduction at 25 μg/mL (Figure 2E). The killing efficiency was reduced when the serum concentration was raised to 5%, although Abp013 still exhibited almost a 3-log CFU reduction at 100 μg/mL. The subsequent increase in the serum concentrations to 10%, greatly reduced the killing activity of Abp013 with a 1.6-log CFU reduction achieved at 400 μg/mL of Abp013. No significant activity was observed at 20% serum concentration.

#### 2.2.5. Host Lytic Spectra of Abp013

**Figure 3:**
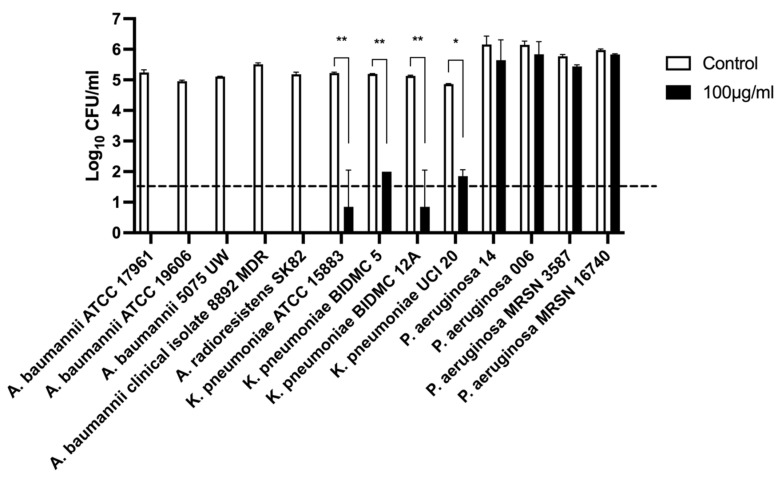
Lytic spectra of Abp013. A total of 4 *A. baumanniii,* 1 *A. radioresistens*, 4 *P. aeruginosa*, and 4 *K. pneumoniae* strains were incubated with 100 μg/mL of Abp013 in 20 mM sodium phosphate buffer, pH 6.0, for an hour at 37 °C. The number of residual log10 CFU/mL was determined through plating 10-fold serial dilution in a bactericidal assay and compared with buffer-treated negative controls. Statistical significance was determined by a two-tailed Student’s *t*-test with Welch’s correction. * *p* < 0.05; ** *p* < 0.01. The dashed line indicates the limit of detection. The experiments were carried out in duplicates; error bars represent the standard deviation.

The spectrum of bactericidal activity of Abp013 was evaluated against a panel of Gram-negative bacteria, including *A. baumannii*, *P. aeruginosa*, and *K. pneumoniae* at 100 μg/mL (Figure 3). Following an hour of incubation, Abp013 was able to eradicate and lyse all the *Acinetobacter* strains, including *A. baumannii* and *A. radioresistens*. Additionally, Abp013 also exhibited effective killing activity against the tested *K. pneumoniae* strains, achieving over 3-log CFU reductions. The same, however, was not observed in *P. aeruginosa*, in which negligible bactericidal activity was observed across all four tested strains.

#### 2.2.6. Activity against *A. baumannii* Biofilm

**Figure 4:**
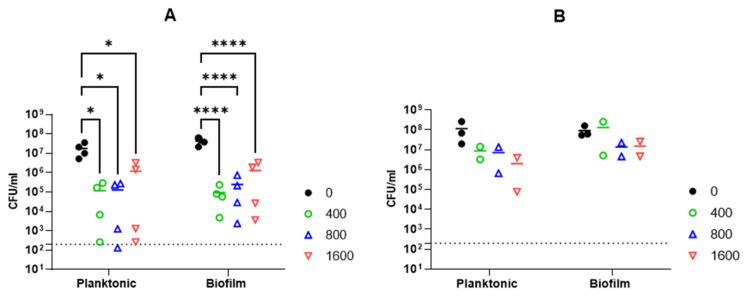
The effect of 3 h treatment of Abp013 on *A. baumannii* ATCC 17961 biofilm that has been pre-grown for 3 h (**A**) or 24 h (**B**). Both planktonic and biofilm cells were collected from the same sample well, with planktonic cells referring to the suspended cells present in the treatment buffer prior to the washing and collection of biofilm cells. Statistical significance was determined using a two-way ANOVA with multiple comparison across treatment groups. * *p* < 0.05; **** *p* < 0.0001. The dashed line indicates the limit of detection. A total of 4 and 2 independent experiments were carried out for 3 h (**A**) and 24 h (**B**) biofilms, respectively, each with 2 technical replicates. Each data point represents the averaged data of an independent experiment.

**Figure 5:**
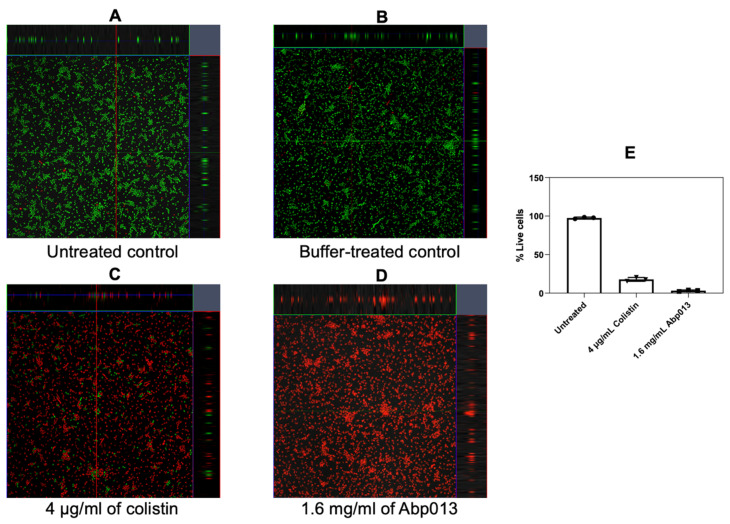
Representative confocal image of untreated control (**A**), buffer-treated control (**B**), 4 µg/mL of colistin (**C**), and 1.6 mg/mL Abp013 (**D**), and the corresponding percentage of live cells (**E**) based on the analysis of at least 3 images for each treatment sample.

The activity of Abp013 was subsequently evaluated in *A. baumannii* ATCC 17961 biofilms. The biofilms were allowed to form for 3 or 24 h before treating with Abp013 in 20 mM of sodium phosphate buffer, pH 6.0, for another 3 h. The growth curve of the biofilm was recorded (Supplementary Figure S3). The initial control test involving the treatment of the 3 h and 24 h biofilm with colistin at 4× MIC was carried out to determine the level of resistance towards antibiotic treatment. The 3 h biofilm showed some levels of resistance towards the colistin treatment, whereas the 24 h biofilm was not eradicated (Supplementary Figure S4). Following Abp013 treatment, 400, 800, and 1600 µg/mL of Abp013 were able to reduce the 3 h biofilm CFU by 2.65-log (99.78%), 2.23-log (99.42%), and 1.51-log (96.93%), respectively, compared to the untreated control (Figure 4A). The activity of the lysin against the 3 h *A. baumannii* biofilms were further verified using confocal microscopy and LIVE/DEAD staining, which indicated that 96.86% of the cells within the biofilm were dead (Figure 5). Figure 4B shows that Abp013 is less effective in treating mature biofilms at a 24 h time point, but higher concentrations of the lysin (800–1600 µg/mL) could nevertheless reduce the 24 h biofilm CFU by 0.827-log (85.13%) and 0.777-log (83.32%). No CFU reduction was observed with 400 µg/mL Abp013 treatment. Planktonic cells collected from the 24 h timepoint were comparatively more susceptible to lysin treatment, and 400, 800, and 1600 µg/mL of Abp013 resulted in a corresponding reduction in CFU by 92.41, 93.80, and 98.27%, respectively.

## 3. Discussion

In this study, we identified and characterized a novel phage lysin Abp013, revealing its natural intrinsic lytic activity against *A. baumannii* and *K. pneumoniae* bacterial strains. Abp013 exhibits the ability to kill the bacterial cells in established biofilms, suggesting the therapeutic potential as an alternative antimicrobial agent against MDR Gram-negative pathogens in clinical settings.

Interestingly, the host range spectra of Abp013 revealed significant lytic activity against *A. baumannii* and *K. pneumoniae*, but not against *P. aeruginosa*. Other than Abp013, Ply6A3 was previously observed to be effective against many *A. baumannii* and *K. pneumoniae* strains, but found to be ineffective against *P. aeruginosa* strains [30]. The absence of the antibacterial activity of Abp013 against *P. aeruginosa* remains largely unknown. However, we ruled out the possibility that the lack of activity against *P. aeruginosa* is due to the catalytic domain of Abp013. Induced lysate clearance assay performed on autoclaved *P. aeruginosa* bacterial agar showed that Abp013 formed a zone of clearance, and thus Abp013 is catalytically capable of hydrolyzing the peptidoglycan of *P. aeruginosa* (Supplementary Figure S5). Therefore, the selective nature of Abp013 likely stems from the cationic C-terminal of Abp013. The differences in the molecular architecture and the diversity of the LPS among Gram-negative bacteria could affect the permeability of the outer membrane, thereby obstructing the entry of Abp013 [18,31]. Understanding such a mechanism requires the structural and dynamic information of the lysin–membrane interactions.

Despite being listed as part of the global priority list by the WHO, few lysins have been reported to be active against *K. pneumoniae* due to its thick capsular polysaccharide layer on the outer cell wall [32]. As such, it is encouraging for Abp013 to exhibit good killing activity against multiple strains of *K. pneumoniae*. As the C-terminal region of Abp013 consists of 9 positively charged residues, we reckon that the positively charged tail of Abp013 may have played a role in anchoring the lysin to the capsular polysaccharide produced by *K. pneumoniae* and the outer membrane of *A. baumannii*, thereby enhancing the permeability.

The pH characterization showed that Abp013 is active between pH 6.0–9.0. Interestingly, Abp013 showed significantly lower activity at pH 7.0. However, we could not pinpoint a mechanism that could explain such an observation. Abp013 is positively charged at pH 7 with an isoelectric point of 9.5, thereby ruling out the possibility of Abp013 crashing out due to a loss of charge. Similar observations were also reported by Wu et al. [30], which saw the increase in the turbidity of Ply6A3 at pH 6.5 and 7.5, but a decrease at pH 7.0. A dose–response assay showed that Abp013 was able to reduce the amount of bacterial cells at 50 μg/mL, below the limit of detection of 50 CFU/mL. Encouragingly, a low lysin concentration required for antibacterial activity would provide a lower production cost for future translation into commercialization [20].

Environmental factors, such as proteins and salts, can significantly influence the lytic activity of Gram-negative lysins. We showed the significant loss of lytic activity of Abp013 in NaCl concentrations above 100 mM, suggesting a low halotolerance. This may be attributed to the electrostatic screening that is imposed by the salt ions that are present in excess. The electrostatic screening thus potentially shields the peptidoglycan layer from Abp013, thereby weakening the electrostatic interaction needed by lysins to bind to the peptidoglycan to exert its lytic capability [33]. Furthermore, in a complex medium, such as human serum, the robust bactericidal activity of Abp013 was observed up until 5% serum, which is better than the results from many previously reported Gram-negative lysins, such as PlyPa03 and PlyE146, with the abolishment of lytic activity observed at 1% serum [34,35]. As Abp013 gradually loses its antibacterial activity above 10% serum concentration, the clinical usage of Abp013 is limited to topical applications, where the direct exposure to serum components is minimal as opposed to a systemic mode of administration [35]. Despite this limitation, the bacteriolytic capacity of Abp013 in low serum conditions offers an exciting possibility of the systemic use of lysins, if the lysins can be further engineered to tolerate higher serum or salt concentrations. Gerstmans et al. [36] reported the production of engineered lysins that are effective in killing several *A. baumannii* strains in up to 90% human serum.

The common observation of the loss of lytic activity imposed by the serum could be explained by the presence of negatively charged molecules found in the serum, resulting in the conjugation and passivation of positively charged residues commonly found in the binding domain of Gram-negative lysins. The resultant neutralized domain would be unable to bind to the negatively charged peptidoglycan, causing the inherent loss of the lysins’ intrinsic ability to permeabilize the outer membrane of Gram-negative bacteria.

The ability for *A. baumannii* to form biofilms aids in its propagation as a widespread nosocomial pathogen [37]. As it has been postulated that the biofilm eradication ability of lysins relies on the ability to kill sessile bacteria that are embedded in the biofilm matrix, the dispersing and destruction of the structural integrity of the bacterial cells could also aid in biofilm destabilization [38]. In this study, the bacteriolytic activity of Abp013 against *A. baumannii* biofilms showed that Abp013 exhibited the ability to gain access and lyse the bacterial cells residing in the biofilm in a dose-dependent manner. While Abp013 was effective in achieving a significant log CFU reduction in the 3 h biofilm, its efficacy was reduced when treating mature biofilms at the 24 h time point, with higher concentrations needed to achieve ~85% of the total CFU reduction. As the growth of biofilms are often dynamic, one determining factor in the outcome of therapeutic regimens is often the age of the biofilms [39]. Older and mature biofilms are known to be less susceptible to antimicrobial agents than younger biofilms and planktonic cells, due to a difference in their metabolic activity and stress response towards antimicrobials [40,41]. This was concordant with our observations that the susceptibility of the biofilm towards Abp013 decreased when the growth time increased from the 3 to 24 h timepoint. Studies conducted by Lood et al. [14] and Raz et al. [35] showed that stationary phase cells were generally more resistant to lysin treatment, which was evident in the significant decrease in activity of Abp013 on the stationary planktonic cells (24 h timepoint), as compared to the 3 h planktonic cells. Interestingly, several studies reporting Gram-negative lysins with an observed efficacy towards biofilm reduction have a different secondary structure make-up from Abp013. For instance, PlyF307 contained a single lysozyme domain and a cationic peptide located in the C-terminal [19], while Abtn-4 contained the glycoside hydrolase family 19 domain and an amphipathic helix at the C-terminal [22].

In summary, we reported Abp013, a novel phage lysin that displays host selectivity, a rare feature in Gram-negative lysins. Unraveling the mechanism behind the lack of killing against *P. aeruginosa* would require future investigations of the structure of Abp013 and its binding dynamics onto various bacterial membranes. These mechanistic understanding will guide protein engineers to design suitable variants with customized selectivity to only targeting the pathogenic bacteria. Given the modular nature of Abp013, it opens the possibility for the efficient swapping of the binding or catalytic domain with other lysins, antimicrobial components, or fusion with antimicrobial peptides, resulting in an engineered chimeric lysin that may display superior antimicrobial potency with improved activity, expanded host spectrum, and robust activity in physiological conditions [19,42].

## 4. Materials and Methods

### 4.1. The Bacterial Strains and Growth Conditions

The bacterial strains utilized in this study are listed in Table 1. The bacterial strains were procured from the American Type Culture Collection (ATCC), Biodefense and Emerging Infections Research Resources Repository (BEI), and the Singapore General Hospital, Department of Microbiology. The bacteria were grown in either liquid broth (shaking at 200 rpm) or solid agar medium consisting of Luria–Bertani (LB) broth at 37 °C. The bacterial strains were suspended in LB broth containing 50% glycerol and maintained at −80 °C for long-term storage.

### 4.2. Bioinformatics Analysis

The nucleotide sequences of Abp013 and 20 others previously reported that Gram-negative lysins were downloaded from the NCBI database and aligned using the ClustalW alignment on the MEGA11 software. These sequences were compared by the Neighbor–Joining method [43]. The bootstrap consensus tree inferred from 1000 replicates was used to represent the evolutionary history of the analyzed taxa [44]. The branches corresponding to the partitions reproduced in less than 50% of the bootstrap replicates were collapsed. The percentage of replicate trees in which the associated taxa clustered together in the bootstrap test were expressed next to the branches [44]. The evolutionary distances were computed based on the p-distance method [45] and expressed as the number of base differences per site. The codon positions included consist of 1st + 2nd + 3rd + noncoding, and all the ambiguous positions were removed for each sequence pair via the pairwise deletion option. There was a total of 989 positions in the final dataset. Evolutionary analyses were conducted in MEGA11 [46]. The functional domain analysis of Abp013 was carried out using the NCBI CDD [47] and SMART [48].

### 4.3. Cloning, Expression, and Purification of Abp013

The Abp013 gene was synthesized and cloned into the pNIC-CH expression vector by Bio Basic Inc. (Markham, ON, Canada). The nucleotide sequences were codon-optimized for the *E. coli* expression system to improve the overall efficacy of the soluble expression. The amino acid sequence for Abp013 can be found in Supplementary Table S2. The recombinant Abp013 fused to a 6× HIS tag at the C-terminal was transformed into BL21(DE3)-T1R *E. coli* competent cells for efficient expression. The transformed cells were incubated in 15 mL of LB broth at 37 °C containing 50 μg/mL of kanamycin and 34 μg/mL of chloramphenicol, and harvested once the mid-log phase of OD_600_ 0.5–0.6 was reached. A total of 5 mL of culture was added to an autoinduction media (47.6 g/L of Terrific Broth, 5 g/L NaCl, 0.15 g/L MgSO_4_, 3.3 g/L (NH_4_)_2_SO_4_, 0.5% glycerol, 0.05% glucose, and 0.2% lactose) containing 50 μg/mL of kanamycin and 34 μg/mL of chloramphenicol; and subsequently incubated at 25 °C for 3 days at 220 rpm. The cells were collected by centrifugation at 4000× *g* for 25 min at 4 °C, and the cell pellet was stored at −80 °C until purification was carried out.

The protein was purified by resuspending the cell pellet with 10 mL of lysis buffer (50 mM HEPES pH 7.5, 500 mM NaCl, 0.5 mM dithiothreitol (DTT), 5% glycerol) for every gram of pellet, and ½ tablet of the Pierce™ protease inhibitor (Thermo Fisher Scientific, Waltham, MA, USA), before disrupting it by sonication on ice. The cell debris was removed via centrifugation at 20,000× *g* for an hour at 4 °C to obtain the soluble proteins. The supernatant was subsequently filtered with a 0.22 μm pore size filter (Sartorius Stedim Biotech, Gottingen, Germany) and incubated with 3 mL of nickel-charged magnetic beads (GenScript, Piscataway, NJ, USA) for 2 h at 4 °C in a tube rotator. The desired proteins were then washed and eluted with lysis buffers containing 0, 10, 20, 250, and 500 mM of imidazole. The purity of the collected fractions was evaluated via sodium dodecyl sulfate-polyacrylamide gel electrophoresis (SDS-PAGE) on a 12% gel and later stained with Coomassie Brilliant Blue-R250. The purified protein fractions were concentrated using a Vivaspin 20 Centrifugal Concentrator 10,000 MWCO PES (Sartorius Stedim Biotech, Gottingen, Germany) to a concentration of >5 mg/mL before storing in aliquots at −80 °C.

### 4.4. Characterization of the Lytic Activities of Abp013

Bactericidal assay was performed in a 96-well microtiter plate to evaluate the lytic activity of Abp013. *A. baumannii* ATCC 17961 was employed in this study as it is a well-characterized reference strain with its genome sequenced [49]. The bacterial culture was inoculated from the LB solid agar medium and grown in 10 mL of LB broth in 37 °C at 200 rpm. The OD_600_ was measured and the bacteria were subcultured into 10 mL of new LB broth at OD_600_ 0.05. Once the early logarithmic phase (OD_600_ 0.3–0.4) bacteria culture was reached, it was centrifuged at 2800× *g* at 16 °C for 10 min, washed twice with 20 mM of sodium phosphate buffer, pH 6.0, and suspended to a final concentration of 10^6^ CFU/mL. To profile the dose–response of *A. baumannii* to Abp013, 10 lysin concentrations ranging from 0.39 μg/mL to 800 μg/mL were diluted in the same buffer and added to the bacteria with an equal volume ratio of 1:1 in a 96-well microplate to a final volume of 100 μL. Upon an hour incubation at 37 °C on an orbital shaker at 200 rpm, the Abp013-treated bacterial cells were serially diluted in 10-folds and plated on a bactericidal assay to determine the minimum inhibitory concentration (MIC). The same volume of buffer was added to the bacterial cells instead of Abp013 as part of the negative control.

To determine the optimal pH condition in which the maximal lytic activity of the lysin could be observed, the reference strain was treated with 100 μg/mL of Abp013. The pH profiling experiment was performed after the bacterial cells were washed and resuspended in 20 mM of sodium phosphate buffer at pH 6.0 and 7.0, and 20 mM of Tris buffer at pH 8.0 and 9.0. Upon setting the pH at 6.0, the further investigation of the lytic activity under NaCl concentrations ranging from 0 to 500 mM in 20 mM of phosphate buffer, pH 6.0, and 1× phosphate-buffered saline (PBS), pH 7.4, was carried out. The influence of the serum on the lytic activity of Abp013 was also studied under varying concentrations of human serum (Sigma Aldrich, St. Louis, MI, USA), ranging from 0% to 20% in 20 mM of phosphate buffer, pH 6.0. A total of 100 μg/mL of Abp013 was chosen to determine the activity and tolerance of Abp013 towards both the salt and human serum.

The lytic activity was measured by the reduction in the log_10_ (CFU/mL) of the viable cells enumerated after incubation with the lysin, as compared to the negative control. All the assays were performed in duplicates and the buffers were autoclaved before use. Data are expressed as the mean ± standard deviation.

### 4.5. Host Range Spectra Determination

The spectrum of the antimicrobial activity of Abp013 (100 μg/mL) was evaluated against a panel of strains listed in Table 1. All bacteria grown to the logarithmic phase were cultured, washed, and resuspended in 20 mM of sodium phosphate, pH 6.0, as determined by the pH characterization. The experiments were carried out in duplicates and data are expressed as the mean ± standard deviation.

### 4.6. Biofilm Assay

Biofilm assay was performed in a 24-well plate to evaluate the lytic activity of Abp013. The overnight cultures of *A. baumannii* ATCC 17961 were diluted in M9 glucose media (1× M9 salts, 2 mM MgSO_4_, 0.1 mM CaCl_2_, 0.4% *w*/*v* glucose) to a final OD_600_ of 0.05. a total of 1 mL of the diluted culture was then added into each well of the 24-well plate and incubated at 37 °C with 100 rpm shaking. Following 3–24 h of incubation, each well was washed once and the culture media was replaced with 1 mL of 20 mM sodium phosphate, pH 6.0, containing 0–1600 µg/mL of Abp013. The treated samples were further incubated under the same conditions for 3 h. At *t* = 6 h or 27 h, the samples were collected. The 20 mM of sodium phosphate (pH 6.0) buffer containing suspended bacteria cells with 0–1600 µg/mL Abp013 treatment was collected into 1.5 mL Eppendorf tubes and considered to be “planktonic samples”. Subsequently, each well was washed once with 1 mL of 20 mM sodium phosphate, pH 6.0, before resuspending the biofilm cells in the same volume of fresh 20 mM sodium phosphate, pH 6.0. The biofilm cells were dislodged into the buffer by means of a cell scraper and 1 mL of the sample was collected into the 1.5 mL Eppendorf tubes and labeled “biofilm samples”. The samples contained in the Eppendorf tubes were sonicated in a water bath using the following settings: 5 min degas mode, 37 Hz, 100%, followed by 5 min pulse mode, 37 Hz, 100%. Subsequently, the samples were serially diluted and used for CFU counts.

### 4.7. Confocal Microscopy

The biofilm was grown and treated, as described above, using a microscopy compatible 24-well plate (µ-plate 24-well black plate, Cat no. 82426, Ibidi). Following the treatment, each well was washed once with 20 mM of sodium phosphate, pH 6.0. Each well was then stained with 200 µL of LIVE/DEAD stain (L7012, Thermo Fisher, Waltham, MA, USA) that was freshly prepared according to the manufacturer’s instructions. Confocal microscopy was carried out using a Carl Zeiss confocal laser scanning microscope LSM 780. Images were obtained using a LD Plan-Neofluar 40×/0.6 dry objective and analyzed using Imaris (Version 8.0.2).

### 4.8. Statistical Analysis

A two-tailed Student’s *t*-test with Welch’s correction was used to evaluate the statistical significance of the obtained results. A *p*-value of <0.05 was considered statistically significant (* *p* < 0.05; ** *p* < 0.01; *** *p* < 0.001). CFU counts in the biofilm assays were analyzed using Graphpad Prism V9.3.0 using 2-way ANOVA and a multiple comparison of column effect (concentration of Abp013) within each row (planktonic vs. biofilm samples).

## Figures and Tables

**Table 1:** Bacterial strains employed in this study.

Strain	Reference/Source
*A. baumannii* ATCC 17961	BEI
*A. baumannii* ATCC 19606	BEI
*A. baumannii* 5075-UW	BEI
*A. baumannii* clinical isolate 8879 MDR	Singapore General Hospital, Department of Microbiology
*A. radioresistens* SK82	BEI
*P. aeruginosa* MRSN 16740	BEI
*P. aeruginosa* MRSN 3587	BEI
*P. aeruginosa* 006	Singapore General Hospital, Department of Microbiology
*P. aeruginosa* 14	BEI
*K. pneumoniae* ATCC 13883	ATCC
*K. pneumoniae* BIDMC 5	BEI
*K. pneumoniae* BIDMC 12A	BEI
*K. pneumoniae* UCI 20	BEI

## Supplementary Materials

The following are available online at https://www.mdpi.com/article/10.3390/antibiotics11020169/s1, Figure S1: sequence alignment of Abp013 with two similar lysins, Figure S2: additional pH profiling, Figure S3: biofilm growth curve of *A. baumannii* ATCC 17961, Figure S4: effect of colistin treatment on early and mature biofilms, Figure S5: induced lysate clearance assay of the *P. aeruginosa* strain PA14, Table S1: domain prediction of Abp013 using SMART analysis, Table S2: amino acid sequence of Abp013 used in this study.
