# Supplementary material for: Novel Phage Lysin Abp013 against Acinetobacter baumannii
Source: Antibiotics (Basel). 2022 Jan 28;11(2):169. doi: 10.3390/antibiotics11020169 (PMC8868305; doi:10.3390/antibiotics11020169)
Supplement: Supplementary file 1 [file antibiotics-11-00169-s001.zip › antibiotics-1524265-supplementary.pdf]

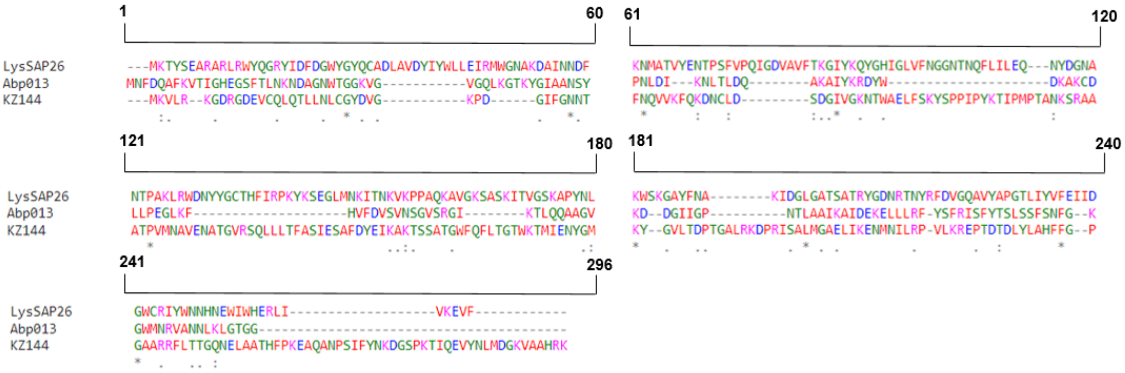

Supplementary Figure S1. Multiple alignment via MUSCLE of Abp013, LysSAP26 and KZ144. Alignment revealed a low similarity between Abp013 and LysSAP26 and KZ144, suggesting the novelty of Abp013.

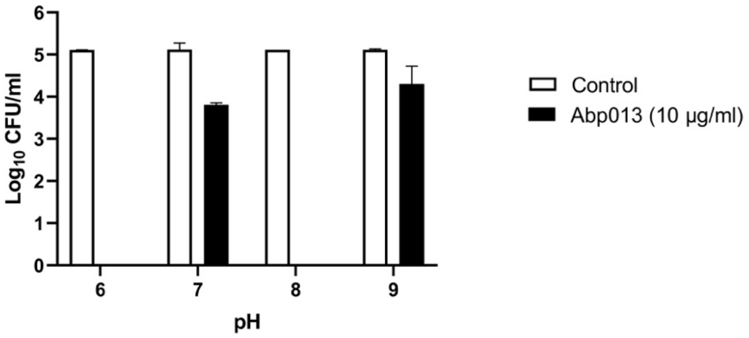

Supplementary Figure S2. Additional pH profiling at a low lysin concentration of 10 μg/ml from pH 6 to 9.

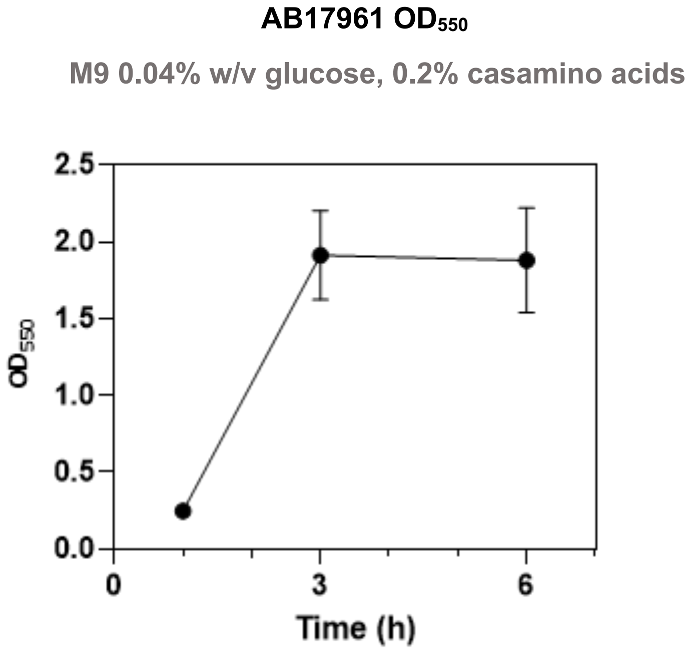

Supplementary Figure S3. Biofilm growth curve of *A. baumannii* ATCC 17961. The biofilm was grown in 24-well plate in M9 medium supplemented with 0.04% w/v glucose and 0.2% w/v casamino acids. The study considered biofilm grown for 3 hours biofilm at 37°C with 120 rpm shaking as young and biofilm grown for 24 hours as more mature biofilms due to the slowing rate of growth after the 8-hour timepoint.

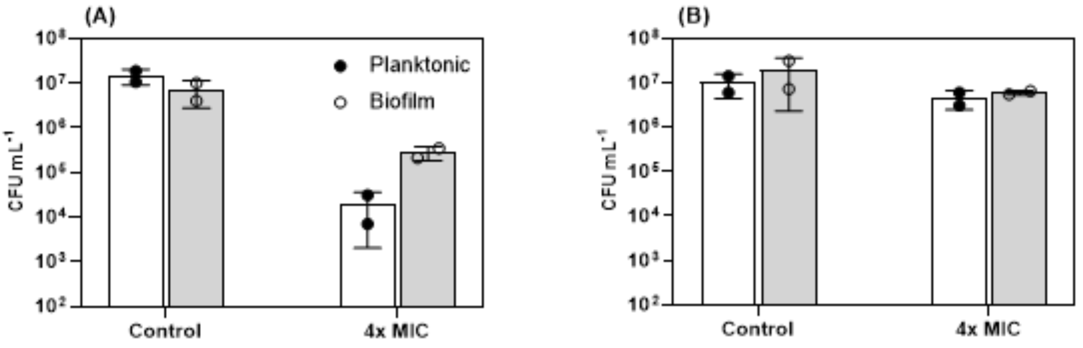

Supplementary Figure S4. Effect of treatment of 3-hour biofilm (A) and 24-hour biofilm (B) with 4 x MIC of colistin. The 3-hour biofilm showed some levels of resistance towards colistin treatment while the 24-hour biofilm showed no levels of eradication by colistin.

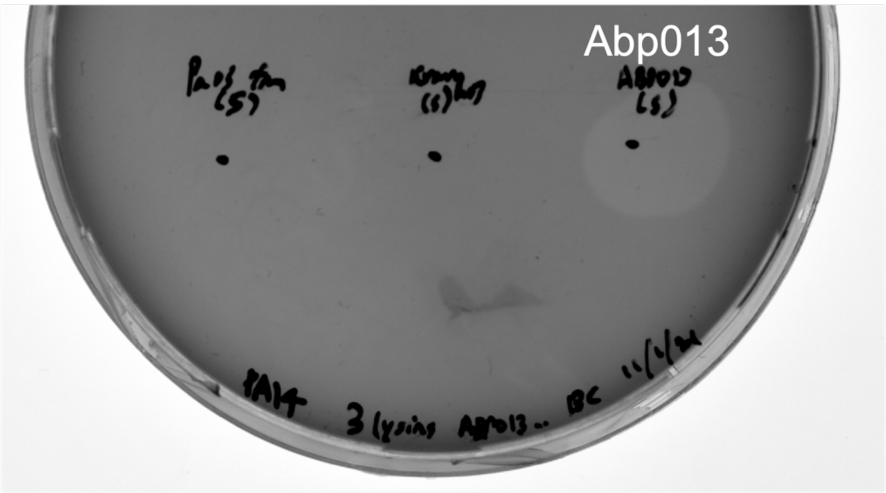

Supplementary Figure S5. Induced lysate clearance assay of *P. aeruginosa* strain PA14. 2.5 µl of lysed induced culture with Abp013 overexpressed was plated onto an agar made of autoclaved PA14 bacteria (right). Zone clearance started to appear after 10 minutes and full zone clearance was observed after 1 hour. As a negative control, the same procedure was done for two unrelated proteins (left and middle dots) but no zone clearance was observed.

Supplementary Table S1. Confidently predicted domains of Abp013 using the Simple Modular Architecture Research Tool (SMART) analysis

Name	Start	End	E-value
Pfam: Glyco_hydro_108	7	95	5.3e-21
Pfam: PG_binding_3	98	161	6.7e-12

Supplementary Table S2. Amino acid sequence of Abp013. The extra 7 residues at the C-terminal (AHHHHHH) is the histidine tag added for purification purposes.

Abp013	MNFDQAFKVTIGHEGSFTLNKNDAGNWTGGKVGVGQLKGTKYGIAANSYP NLDIKNLTLDQAKAIYKRDYWDKAKCDLLPEGLKFHVFDVSVNSGVSRGIKTL QQAAGVKDDGIIGPNTLAAIKAIDEKELLLRFYSFRISFYTSLSSFSNFGKGWMN RVANNLKLGTGGAHHHHHH
